# Each Mycobacterium Requires a Specific Culture Medium Composition for Triggering an Optimized Immunomodulatory and Antitumoral Effect

**DOI:** 10.3390/microorganisms8050734

**Published:** 2020-05-14

**Authors:** Sandra Guallar-Garrido, Víctor Campo-Pérez, Alejandro Sánchez-Chardi, Marina Luquin, Esther Julián

**Affiliations:** 1 Departament de Genètica i de Microbiologia, Facultat de Biociències, Universitat Autònoma de Barcelona, Bellaterra, 08193 Barcelona, Spain; sandra.guallar@uab.cat (S.G.-G.); victor.campo@uab.cat (V.C.-P.); marina.luquin@uab.cat (M.L.); 2 Bacterial Infections and Antimicrobial Therapies group, Institute for Bioengineering of Catalonia (IBEC), The Barcelona Institute of Science and Technology (BIST), 08028 Barcelona, Spain; 3 Servei de Microscòpia, Universitat Autònoma de Barcelona, 08193 Barcelona, Spain; alejandro.sanchez.chardi@uab.cat; 4 Departament de Biologia Evolutiva, Ecologia i Ciències Ambientals, Facultat de Biologia, Universitat de Barcelona, 08028 Barcelona, Spain

**Keywords:** BCG, *M. brumae*, nonmuscle invasive, bladder cancer, Sauton, Middlebrook

## Abstract

*Mycobacterium bovis* bacillus Calmette-Guérin (BCG) remains the first treatment option for non-muscle-invasive bladder cancer (BC) patients. In research laboratories, *M. bovis* BCG is mainly grown in commercially available media supplemented with animal-derived agents that favor its growth, while biomass production for patient treatment is performed in Sauton medium which lacks animal-derived components. However, there is not a standardized formulation of Sauton medium, which could affect mycobacterial characteristics. Here, the impact of culture composition on the immunomodulatory and antitumor capacity of *M. bovis* BCG and *Mycolicibacterium brumae*, recently described as efficacious for BC treatment, has been addressed. Both mycobacteria grown in Middlebrook and different Sauton formulations, differing in the source of nitrogen and amount of carbon source, were studied. Our results indicate the relevance of culture medium composition on the antitumor effect triggered by mycobacteria, indicating that the most productive culture medium is not necessarily the formulation that provides the most favorable immunomodulatory profile and the highest capacity to inhibit BC cell growth. Strikingly, each mycobacterial species requires a specific culture medium composition to provide the best profile as an immunotherapeutic agent for BC treatment. Our results highlight the relevance of meticulousness in mycobacteria production, providing insight into the application of these bacteria in BC research.

## 1. Introduction

The bacterium *Mycobacterium bovis* bacillus Calmette-Guérin (BCG) has been produced as an immunotherapeutic agent for almost 100 years. *M. bovis* BCG has two main therapeutic uses: as a preventive vaccine for tuberculosis and as a therapeutic agent for non-muscle invasive bladder cancer (NMIBC) patients. For both uses, *M. bovis* BCG production is relevant. In the case of tuberculosis, *M. bovis* BCG has been used from the beginning of the 19th century and remains the only available vaccine for the prevention of the most deadly disease worldwide [1], making *M. bovis* BCG the most widely used live vaccine [2]. In the case of bladder cancer (BC) treatment, *M. bovis* BCG is the preferred treatment option in NMIBC patients [3]. BC was considered the seventh most frequent type of cancer detected in the male population in 2016, and the 11th when the statistics included women [4]. Approximately 75% of the diagnosed cases of BC are NMIBC. For these patients, weekly intravesical instillations of *M. bovis* BCG after transurethral resection of the tumor are carried out to prevent recurrence and progression of the disease. Recent shortages in *M. bovis* BCG production have highlighted the crucial role of mycobacteria in the treatment of these patients.

Although the precise mechanism underlying its antitumor efficacy is not completely known, in vitro and in vivo studies have demonstrated that *M. bovis* BCG triggers a direct antitumor effect by inhibiting BC cell proliferation and has an indirect immunotherapeutic effect by attracting circulating immune cells into the bladder, which release cytokines and induce a cytotoxic profile that resolves the tumor growth. Unfortunately, *M. bovis* BCG treatment has adverse effects, ranging from mild reactions to infections, in a high percentage of patients [5,6]. Moreover, the recent supply shortages of *M. bovis* BCG worldwide have highlighted its costly production, due mainly to its slow growth. In the search for *M. bovis* BCG alternatives, our group has demonstrated both the direct and indirect antitumor effect of the non-tuberculous mycobacterium sp. *Mycolicibacterium brumae* (basonym *Mycobacterium brumae*) (Appendix A) [7,8,9]. Intravesical instillation of *M. brumae* in tumor-bearing mice triggers survival rates similar to those observed for *M. bovis* BCG, demonstrating the capacity of this species to activate the immune response in a manner safer than that observed with *M. bovis* BCG. Furthermore, *M. brumae* grows three times faster than BCG and is therefore faster and cheaper to produce than *M. bovis* BCG.

In the context of tuberculosis prevention, the relationship between the immunotherapeutic ability of *M. bovis* BCG and the culture media in which *M. bovis* BCG grows is discussed. A majority of the research laboratories that study the effects of *M. bovis* BCG utilize commercially available Middlebrook culture media supplemented with glycerol, bovine serum albumin, catalase, oleic acid, and dextrose. However, for vaccine production, *M. bovis* BCG is mainly grown as a surface pellicle on animal-component-free Sauton medium [10,11]. Sauton medium was developed in 1912, and different formulations are available worldwide (Supplementary Table S1) [12,13,14,15,16,17,18,19,20]. The main differences in the formulation of Sauton medium lie in the source of amino acids (L-asparagine or L-glutamate), the glycerol concentration used as a carbon source, and the presence or absence of zinc sulphate. Several studies on the characteristics of *M. bovis* BCG grown in Middlebrook and Sauton media revealed that the protein/antigen expression profiles, the resistance to reactive nitrogen intermediates [21], and the triggered humoral immune response in immune cells in vitro or in mice (including protection against *Mycobacterium tuberculosis* challenge in mice) [11] are modified as a function of the culture medium used for *M. bovis* BCG growth. However, the impact of culture medium on the antitumor effect of *M. bovis* BCG has not been examined previously. Furthermore, nothing is known about the influence of culture media on other potential immunotherapeutic agents, such as *M. brumae*. The presence of zinc sulphate, the concentration of glycerol, or the amino acid source could influence in the growth of *M. brumae* as it has been described for *M. bovis* BCG and other mycobacteria [20,22,23,24]. Therefore, we aimed to determine the effect of growth in four modified Sauton formulations and in liquid and solid Middlebrook media on the antitumor ability of two mycobacteria. The direct antitumor effect of mycobacteria was studied on five different BC cell cultures, and the immunomodulatory effect was studied on two different macrophage cell lines.

## 2. Materials and Methods 

### 2.1. Preparation of Different Culture Media

Liquid Sauton media were prepared by sequentially adding, with constant agitation, the following compounds: citric acid (Fluka Chemika, Steinheim, Germany) 2 g/L, potassium dihydrogen phosphate (Panreac, Barcelona, Spain) 0.5 g/L, ferric ammonium citrate (Sigma-Aldrich, St. Louis, USA) 0.05 g/L, magnesium sulphate heptahydrate (Fluka Chemika) 1 g/L, zinc sulphate (Panreac), and 2–4 g/L L-asparagine (Scharlau, Sentmenat, Spain) or L-glutamate (Panreac). Finally, glycerol (from 15 to 60 mL/L) was added (Panreac), and the pH was adjusted to 7.2–7.3. All media were sterilized by autoclaving for 15 min at 121 °C. Middlebrook 7H10 medium (Becton and Dickinson, Le Pont de Claix, France) supplemented with 10% oleic albumin dextrose catalase enrichment medium was prepared as described previously [7], and Middlebrook 7H9 medium (Becton and Dickinson, Le Pont de Claix, France) was supplemented with 10% albumin dextrose catalase enrichment medium.

### 2.2. Bacterial Strains and Culture Conditions

*M. bovis* BCG Connaught (ATCC 35745) and *M. brumae* (ATCC 51384^T^) were initially grown on Middlebrook 7H10 at 37 °C for 4 weeks and 1 week, respectively. Mycobacterial colonies were scraped from 7H10 medium, suspended in phosphate-buffered saline (PBS) and adjusted to a 1.0 McFarland turbidity standard [25]. The same amount of mycobacteria (3 × 10^4^ colony forming units (CFU)/mL) was carefully added on the surface of 50 mL of each medium. Cultures were incubated in static conditions for 28 or 11 days for *M. bovis* BCG or *M. brumae*, respectively. After incubation, the pellicle formed was recovered under sterile conditions for further analyses or filtered, dehydrated, and weighed to calculate biomass production.

### 2.3. Preparation for Ultrastructural Assessment of Mycobacterial Pellicles and Bacilli 

Pellicles were collected with Nucleopore™ membranes (Whatman^®^, Maidstone, UK), located in Nunc 6-well plates, (Thermo Fisher Scientific, Roskilde, Denmark) and fixed with osmium vapors with 2 mL of 4% osmium tetraoxide (TAAB Lab., Aldermaston, UK) at 4 °C overnight. Samples were then air dried during 24 h at room temperature, placed in metallic stubs with carbon adhesive discs, and coated with Au-Pd. Images of pellicles were analyzed with an scanning electron microscope (SEM) EVO MA 10 (Zeiss, Oberkochen, Germany) equipped with a standard secondary electrons detector and operating at 15 kV. For each pellicle from duplicate experiments, between three and four representative SEM images of intact pellicle microarchitecture were obtained from randomly selected fields for the qualitative study. For the quantitative study of bacilli morphology, the size of 50 entire cells of each condition were quantified with the length of major axis of bacilli from the top of the cords by two blind evaluators and from duplicate experiments using ImageJ software [26,27,28,29].

### 2.4. Cell Culture Conditions

SW780, 5637, and T24 human BC cell lines were obtained from the Cancer Cell Line Repository (RTICCC-PRBB) and were maintained in Dulbecco’s modified Eagle’s medium (DMEM)/Ham’s F12 nutrient mixture (Gibco, Paisley, UK); J774A.1 cell line (DSMZ ACC 170) and the murine MB49 tumor bladder cells, kindly provided by Dr. Mangsbo and Dr. Tötterman from Rudbeck Laboratory, Uppsala University, Sweden, were maintained in DMEM with stable L-glutamine (Gibco); and human THP-1 macrophages (DSMZ ACC 16) were maintained in RPMI medium (Lonza, Walkersville, USA). All media were supplemented with 10% fetal bovine serum (Lonza), containing 100 U/mL penicillin G (Laboratorios ERN, Barcelona, Spain) and 100 g/mL streptomycin (Laboratorio Reig Jofré, Barcelona, Spain) (complete media).

### 2.5. Tumor Growth Inhibition Experiments

Growth inhibition experiments were performed as previously described [8,30,31]. Briefly, *M. brumae* or *M. bovis* BCG cells were taken in sterile conditions with a Kollë handle from the bottom part of the pellicle to the top. Then, mycobacteria were disaggregated with glass beads and a suspension similar to McFarland 1 standard was performed. Bacteria concentration was corroborated by culturing in Middlebrook 7H10 medium for CFU counts (Supplementary Table S2). Tumor cells (3 × 10^4^) were seeded into 96-well tissue culture plates (Nunc) and infected with *M. bovis* BCG or *M. brumae* (MOI 10) grown in different culture media. Three hours later, extracellular mycobacteria were removed by washing with warm PBS. Complete medium was then added, and cells were incubated for 72 h. After incubation, supernatants were collected, centrifuged and stored at −80 °C for further cytokine analysis. Tumor cell viability was then performed using a 3-(4,5-dimethylthiazol-2-yl)-2,5-diphenyltetrazolium bromide (MTT) colorimetric assay (Sigma-Aldrich, St. Louis, USA).

### 2.6. Mycobacterial Survival inside Macrophages

THP-1 cells were seeded at 8.5 × 10^4^ cells/well into 48-well tissue culture plates (Nunc) and differentiated into macrophages by adding phorbol 12-myristate 13-acetate (PMA, Abcam, Cambridge, UK) at 100 nM for 48 h. Then, PMA was removed, and new media was added for 24 h. J774 cells were seeded in 48-well tissue culture plates (Nunc) at 6 × 10^4^ cells/well and incubated for 3 h at 37 °C. Both THP-1 and J774 macrophages were infected with *M. bovis* BCG or *M. brumae* (MOI 1 or 10, respectively) grown in different culture media as described above and incubated at 37 °C. At different time points (3, 24, 48, 72, 96, and 120 h), supernatants were collected and stored, and cells were lysed by adding 0.1% Triton X-100 (Sigma-Aldrich). Colony forming unit (CFU) counts of intracellular mycobacteria were determined by plating serial dilutions of the lysate on Middlebrook 7H10 plates.

### 2.7. Cytokine Analysis

The presence of cytokines and chemokines in culture supernatants was detected using commercially available enzyme immunoassays following the manufacturer’s instructions. Human IL-12, TNFα, and IL1β and mouse IL-12 and TNFα were detected using Mabtech (Nacka Strand, Sweden) tests, and human IL-6 and CXCL-8 and mouse IL-6 were detected using Becton and Dickinson (BD, San Diego, USA) tests. KC (a mouse cytokine homologous to CXCL-8) was evaluated using a R&D Systems test (R&D Systems, Minneapolis, USA). IL-6, IL-1β, and NO were analyzed at 72 h, while TNF-α and IL-12 were measured at 96 h.

### 2.8. Statistics

All experiments were performed at least three times. The data represent the mean ± standard deviation (SD) of three independent pellicles per condition. Student’s *t*-test was used to assess the statistical significance of differences for biomass production in presence of zinc sulphate in Sauton media. Analysis of variance (ANOVA) with Bonferroni post-test was used to assess the significance of influence of amino acid source and glycerol concentration in biomass production, *M. brumae* and *M. bovis* BCG length, mycobacteria survival inside macrophages, antitumor activity on tested cell lines, and cytokine levels produced by macrophages and bladder cell lines. Analyses were performed using GraphPad Prism version 6 (San Diego, CA, USA). Statistical significance was considered at *p <* 0.05.

## 3. Results

### 3.1. Presence of ZnSO_4_ and L-Asparagine and Increasing Glycerol Concentrations in Culture Media Determine the Growth of *M. brumae*

Based on previously described formulations for *M. bovis* BCG production (summarized with representative references for each medium in Supplementary Table S1), different Sauton formulations were designed to study for the first time the growth of *M. brumae* in animal-component-free culture media. Most of the components were constant, but the presence of ZnSO_4_, the concentration of glycerol, and the source of nitrogen used varied among the different reported media. We then analyzed the influence of each of these components on *M. brumae* growth. 

**Figure 1:**
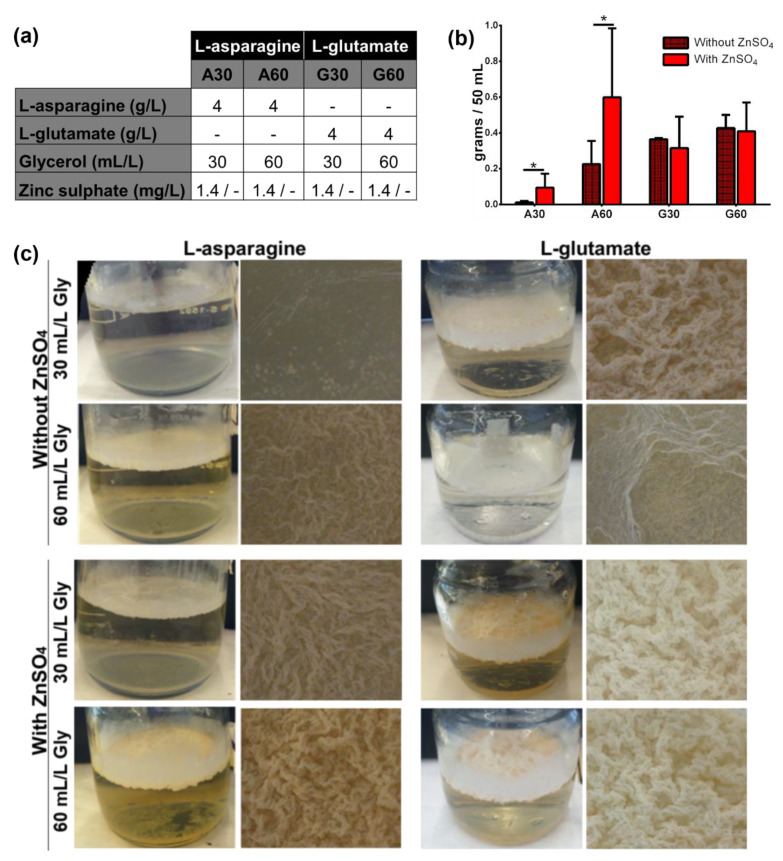
Influence of the presence of zinc sulphate in Sauton media on *Mycolicibacterium brumae* growth in pellicles. (**a**) Culture medium composition for evaluating the influence of ZnSO_4_ on *M. brumae* growth. Each medium was evaluated in the presence of ZnSO_4_ (1.4 mg/L) or in its absence (-); (**b**) biomass production of *M. brumae* pellicles grown in presence or absence of ZnSO_4_; (**c**) macroscopic appearance. Data represent the mean ± standard deviation (SD) in each medium from at least three independent experiments. * *p* < 0.05 (Student’s *t-*test).

The impact of the presence of ZnSO_4_ was initially evaluated using eight different formulations (Figure 1a). Macroscopic appearance of *M. brumae* pellicles clearly correlates with biomass production. Flat, thin, breakable, and partly folded pellicle was observed when mycobacteria grew in the absence of ZnSO_4_ and low concentration of glycerol (Figure 1c). However, *M. brumae* pellicle became rough, robust, and densely folded when mycobacteria grew in culture medium with ZnSO_4_, showing increasing features when high glycerol concentration is present. Moreover, in asparagine-containing media with ZnSO_4_, a significant increase in biomass production together with a robust mycobacterial pellicle formation was observed (Figure 1b,c). 

In contrast, biomass production was independent of the presence of ZnSO_4_ in L-glutamate-containing media, not improving the consistency of the *M. brumae* pellicle (Figure 1b,c).

**Figure 2:**
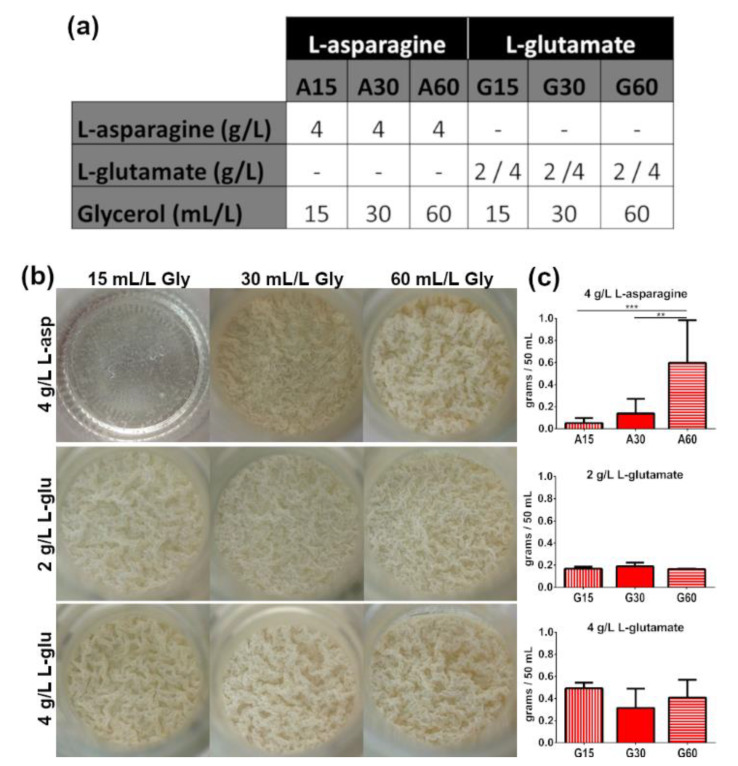
Influence of the amino acid source and glycerol concentration in Sauton medium on the growth of *M. brumae* in pellicles. (**a**) Formulation of the different Sauton media used in the study. L-glutamate was tested at two different concentrations (2 and 4 g/L); (**b**) macroscopic appearance; (**c**) biomass production of *M. brumae* pellicles grown in different amino acid sources and glycerol concentrations. Data represent the mean ± SD in each culture medium from at least three independent experiments. ** *p <* 0.001; *** *p <* 0.0001 (ANOVA test).

Therefore, the presence of ZnSO_4_ was fixed in the media compositions, and the influence of the source of amino acids and glycerol concentrations was evaluated (nine different conditions in Figure 2a). In L-asparagine-containing media, a correlation between *M. brumae* biomass production and glycerol concentration was observed (Figure 2c), as reflected in the macroscopic morphology of the cultures (Figure 2b). In contrast, the concentration of glycerol in the formulation did not affect the growth of *M. brumae* when L-glutamate was used as an amino acid source. As shown in Figure 2b, similar pellicles were observed in L-glutamate-containing media independent of the glycerol concentration, with increased growth of *M. brumae* detected when the highest amount of L-glutamate was used in the formulation (Figure 2b,c). Sauton A60 was the medium that produced the highest *M. brumae* biomass, followed by high-dose L-glutamate-containing media. A60, G15, and G60 were selected for further analysis.

### 3.2. Culture Composition Differentially Influences *M. brumae* and *M. bovis* BCG Biomass Production but Not in Terms of Microscopic Appearance

**Figure 3:**
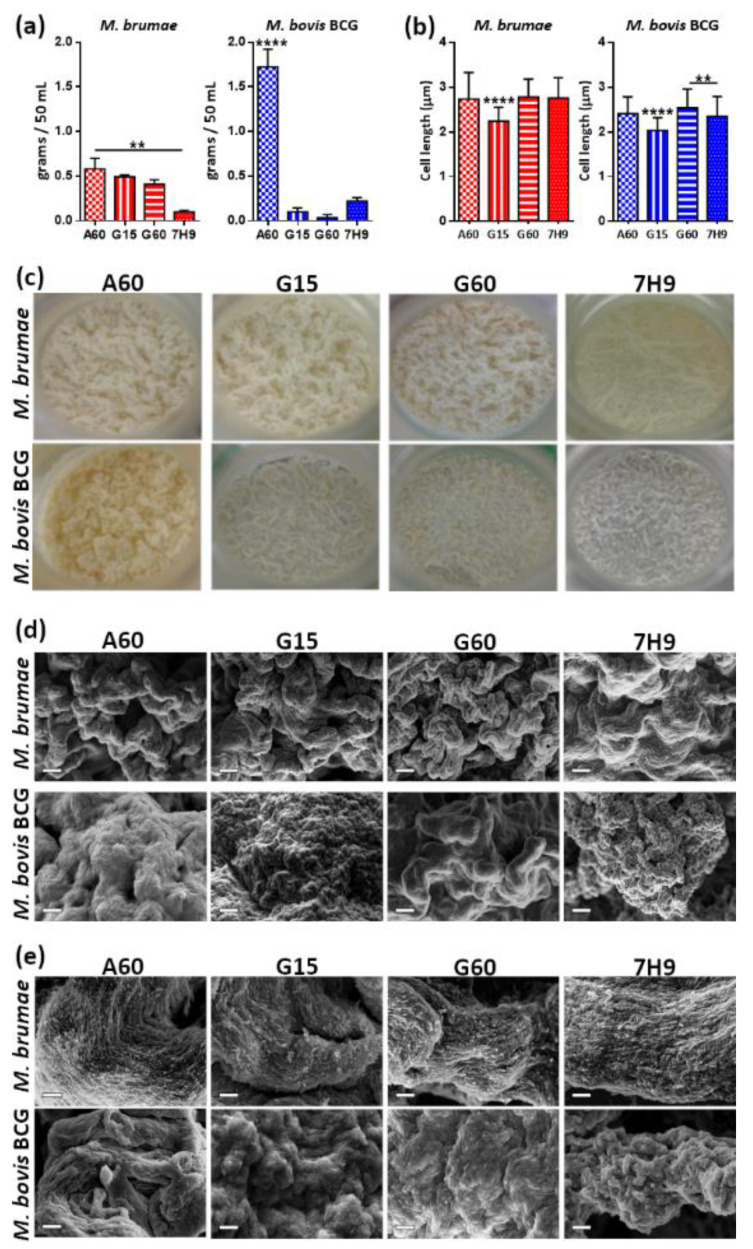
Characteristics of mycobacteria grown in optimized Sauton media. (**a**) Biomass production and (**b**) length of *M. brumae* and *Mycobacterium bovis* bacillus Calmette–Guérin (BCG) cells in each culture medium. Length is expressed in micrometres (μm). Data represent the mean ± SD in each medium from at least three independent experiments. ** *p <* 0.01, *** *p <* 0.001 (ANOVA test). (**c**) Macroscopic appearance and (**d,e**) representative SEM micrographs of *M. brumae* and *M. bovis* BCG pellicles in each culture medium. The bar size is 30 µm in (**d**) and 6 µm in (**e**).

When the growth of *M. brumae* and *M. bovis* BCG on different Sauton formulations was compared with that on 7H9 medium, at least one Sauton formulation exhibited higher biomass production than 7H9 medium. In the case of *M. bovis* BCG, biomass production on A60 culture medium was particularly significantly higher than that observed with the rest of the media (Figure 3a). Mycobacterial length was also altered as a function of the medium on which the cells were grown. In both cases, for *M. brumae* and *M. bovis* BCG, the average major axis length of the cells was significantly lower in G15 culture medium than in the other media, as shown in Figure 3b. When a macroscopic analysis was conducted, *M. brumae* cultures grown on Sauton media and *M. bovis* BCG grown on A60 had a similar appearance, showing solid raised wrinkles and extremely robust and consistent pellicle. Contrary, *M. brumae* grown in 7H9 or *M. bovis* BCG grown in the rest of the media appeared as flat, thin, fragile, and breakable pellicles. Those results are according to the biomass produced (Figure 3b).

No significant differences among bacteria grown in different culture media were observed for either *M. bovis* BCG or *M. brumae* through SEM analysis, although different appearances were observed between the mycobacterial *M. brumae* and *M. bovis* BCG pellicles (Figure 3d,e and Supplementary Figures S1 and S2). *M. brumae* showed free cells grouped with an evident directionality, presenting infinity of folds that imparted an abundant roughness. *M. bovis* BCG exhibited a thick extracellular matrix that covered the outermost surface of the pellicle, giving a compact appearance, and bacteria were present under this layer; in addition, this pellicle exhibited low roughness and few folds. 

### 3.3. Culture Conditions Affect the Immune Response Triggered by Mycobacteria but Do Not Modify Their Survival in Macrophages

**Figure 4:**
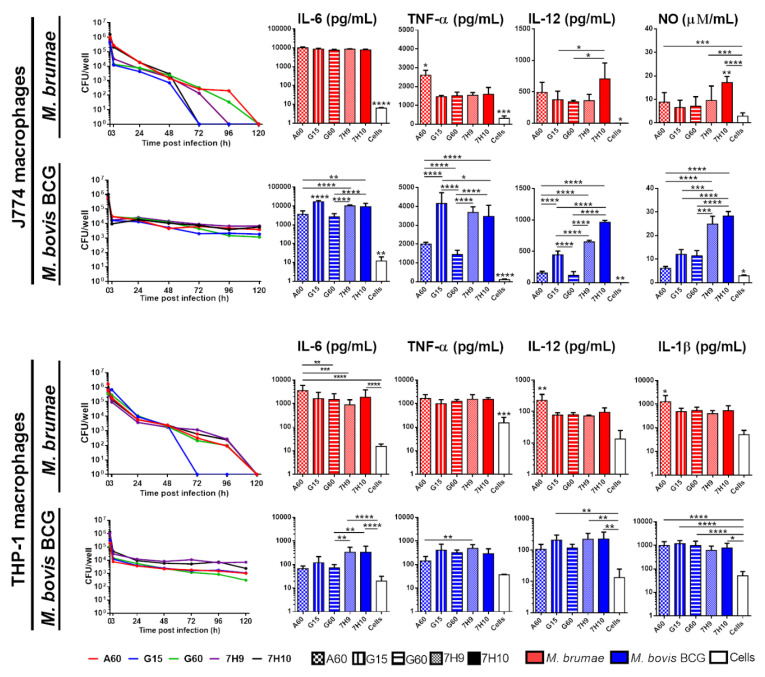
Mycobacterial survival rates and cytokine production triggered by macrophages infected with *M. brumae* or *M. bovis* BCG grown on different culture media. Mycobacteria-infected J774 and THP-1 macrophages were lysed at different time points after infection, and mycobacterial colony forming units (CFUs) inside macrophages were counted. Graphs show the mycobacterial burden at different time points. Cytokine production and NO was evaluated in culture supernatants collected 72 h (NO, IL-12, and TNF-a) and 96 h (IL-1β and IL-6) after mycobacterial infection using commercially available ELISA tests. *M. brumae* in red and *M. bovis* BCG in blue. Data represent the mean ± SD from three independent experiments. * *p <* 0.05; ** *p <* 0.01; *** *p <* 0.001; ****, *p <* 0.0001 (ANOVA test).

Similar survival of *M. brumae* inside J774- and THP-1-infected macrophages was observed independently of the culture medium used for *M. brumae* growth (Figure 4). Both macrophage cell lines were able to kill *M. brumae* in 72 h (when *M. brumae* is grown in G15 and 7H10) and 120 h (for the rest of conditions) after infection, without significant differences between them. Although macrophages do not permit the survival of *M. brumae*, the induction of an immune reaction was clearly influenced by the growth medium. *M. brumae*-A60 induced significantly higher production of TNF-α and IL-12 in J774 and significantly higher production of IL-6, TNF-α, IL-12, and IL-1β in THP-1 than those observed in other conditions. 

In contrast, the number of viable *M. bovis* BCG cells decreased in the first 3 h and then remained steady at only one order of magnitude lower than the infection dose in both macrophage cell lines. Three hours after infection, CFU levels of *M. bovis* BCG diminished from an average of 7.2 × 10^5^ to 2.3 × 10^4^ CFU/mL and 7.8 × 10^5^ to 2.1 × 10^4^ CFU/mL, in THP1 and J774 macrophages, respectively (Figure 4). In THP-1 macrophages, the Middlebrook-grown *M. bovis* BCG burden remained higher than the Sauton-grown *M. bovis* BCG burden, although this difference was not statistically significant. G15-BCG and Middlebrock-grown *M. bovis* BCG induced higher cytokine production than *M. bovis* BCG grown in the rest of the Sauton formulations, with significantly high levels of IL-6, TNF-α, IL-12, and IL-1β production in J774 cell and IL-12 production in THP-1 cells.

### 3.4. Antitumor Activity is Enhanced in L-Asparagine-Containing Medium for *M. brumae* and in L-Glutamate-Containing Media for *M. bovis* BCG

**Figure 5:**
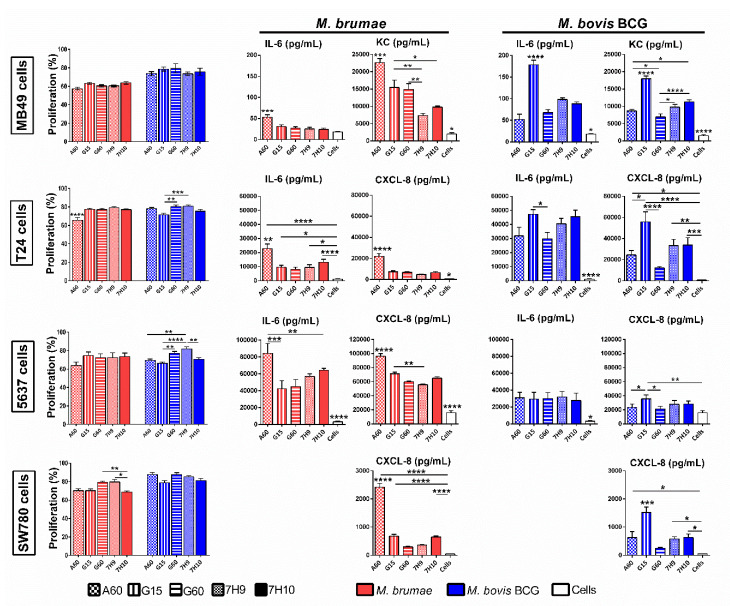
Cell growth inhibition and cytokine production by *M. brumae* and *M. bovis* BCG-infected bladder cancer (BC) cell lines. The antitumor effect of *M. brumae* (in red) and *M. bovis* BCG (in blue) grown in different culture media was evaluated on murine (MB49) and human (T24, 5637, and SW780) BC cell lines. Graphs show the percentage of growth inhibition with respect to non-infected cells using the 3-(4,5-dimethylthiazol-2-yl)-2,5-diphenyltetrazolium bromide (MTT) colorimetric assay. Cytokine production in cell culture supernatants collected 72 h after mycobacterial infection was evaluated using ELISA tests. Data represent the mean ± SD from at least three independent experiments. * *p <* 0.05; ** *p <* 0.01; *** *p <* 0.001; **** *p <* 0.0001 (ANOVA test).

When inhibition of tumor proliferation was measured, *M. brumae* grown in A60 medium exhibited the highest antiproliferative capacity compared to *M. brumae* grown in the rest of culture media (Figure 5). This higher growth inhibition is correlated with significantly higher cytokine production (IL-6 as well as KC or CXCL-8) compared to that of *M. brumae* grown in L-glutamate-containing media or Middlebrook (Figure 5).

The antitumor effect of *M. bovis* BCG depends on the cell line analyzed. However, G15-BCG tended to inhibit BC cells more than *M. bovis* BCG grown in the other media (Figure 5). G15-BCG-triggered cytokine production was also significantly higher than that induced by *M. bovis* BCG grown in the rest of the media. Only in the case of 5637 BC cells, similar IL-6 production was observed among all tested conditions. Middlebrook-grown *M. bovis* BCG triggered a similar pattern of cytokine production, but 7H9-BCG had a weaker antiproliferative effect on BC cells (Figure 5).

## 4. Discussion

In our previous studies in which *M. brumae,* with immunotherapeutic activity, was described as an agent for cancer treatment [7,8,9], the strain was cultured on commercially available Middlebrook 7H10 medium. Middlebrook media (7H9, which is the liquid form, and 7H10/7H11, the solid form) are traditional culture media used to grow fastidious mycobacteria because they are enriched with bovine serum albumin, among other components that facilitate their growth. In fact, a vast majority of research laboratories use these media for growing mycobacteria. However, when mycobacteria must be grown for biomass production, animal-component-free culture media are required. Sauton is the medium of choice for *M. bovis* BCG production in the biotechnological context, but there is not a unique standardized Sauton formulation [2,15,24,32,33]. In our work, different Sauton formulations were assayed to optimize *M. brumae* production.

Our results first demonstrate that the presence of zinc sulphate is essential to obtain optimal growth of *M. brumae* when L-asparagine is the amino acid source in the formulation, with no impact observed when L-asparagine was substituted with L-glutamate. Accordingly, the presence of this inorganic compound in cultures of *M. tuberculosis* provides improved biomass production and it plays an important role in media with asparagine as a nitrogen source [34]. This could be explained by the presence of the aspartate transaminase enzyme, which produces L-glutamate from aspartate, obtained from asparagine hydrolysis, which is more efficient in the presence of zinc, allowing the formation of larger pellicles, as described for Sauton-grown *M. bovis* BCG [23,35]. The addition of zinc sulphate was therefore included in *M. brumae* culture optimization.

The results demonstrated that *M. brumae* was able to grow using both L-asparagine and L-glutamate as sources of nitrogen, reaching a similar biomass at the same concentration of the substrate. However, *M. bovis* BCG cultures produced significantly higher amounts of biomass when L-asparagine was employed. The metabolism of nitrogen is complex in mycobacteria. Although most works have described that mycobacteria prefer L-asparagine to L-glutamate [36,37,38], differences in growth kinetics and final biomass between *M. tuberculosis* and *M. smegmatis* have been described in identical culture media, indicating species-specific variations in nitrogen metabolism [39]. Differences in nitrogen metabolism could be due to the lack of functional metabolic enzymes in some species. In fact, such differences have been described even among *M. bovis* BCG sub-strains [40]. Another influential factor could be the presence of sensing mechanisms on the mycobacterial surface to control nutrient uptake, which could drive both mycobacteria toward a variable capacity to assimilate each substrate based on the constitutive or inducible expression of these sensors, as has been described in *M. tuberculosis* and other mycobacteria [36,40,41,42]. Moreover, the source of carbon in the culture medium also influences mycobacterial central metabolism [42,43]. In fact, *M. bovis* BCG can use L-glutamate as both a nitrogen and carbon source [44] in addition to the glycerol added to the medium. In *M. tuberculosis*, the simultaneous co-catabolism of two different carbon sources via complimentary routes [45] has been described but does not improve the growth of mycobacteria [39]. Further research is needed to analyze the pairing between nitrogen sources and glycerol as a carbon source related to biomass production.

When the interaction between mycobacteria and eukaryotic cells was studied, our results demonstrated that the composition of the culture medium has an impact on the antitumor and immunostimulatory activity of mycobacteria. *M. brumae* and *M. bovis* BCG enter both macrophages and BC cells and are killed or not (*M. brumae* or *M. bovis* BCG, respectively) independently of the medium in which they were grown. However, the capacity to inhibit tumor growth and to trigger a favorable immune response was significantly enhanced in *M. brumae* grown in A60 and in *M. bovis* BCG grown in G15 compared to the rest of the culture conditions. We hypothesize that the differences observed between both mycobacteria could be explained by a different antigenic profile, which could clearly influence the interaction with the host. In fact, growing evidence has suggested direct links between metabolic adaptation of mycobacteria and the composition and immunoreactivity of their cell surface lipids [46]. In response to the environment, mycobacteria are able to control the composition and quantity of immunostimulatory molecules. It is known that growth in glycerol-rich medium leads to increased amounts of lipids and glycolipids on the mycobacterial cell wall, as has been described, for instance, in *Mycobacterium phlei* or *Mycobacterium avium* subsp. *hominissuis* [24,27]. This may account for the high mycobacterial cell length found in our work (Figure 3) and previous works [47] or/and the high mass of bacilli obtained on this substrate per unit volume of culture medium [24]. Glycerol assimilation can alter growth rate, metabolism, cellular structure, and even drug sensitivity in mycobacteria [48]. After entering cells via membrane diffusion [49], glycerol drives catabolism, allowing mycobacterial cells to obtain ATP, or anabolism, triggering high levels of cell wall compounds such as trehalose mono- and diacyl-mycolates or triacylglycerides [50,51] that are involved in interactions with host cells [52,53,54]. Although the cell wall composition differs substantially between *M. brumae* and *M. bovis* BCG [55], these mycobacteria share several genus-specific antigens, such as the previously mentioned trehalose-mycolates or phosphatidylinositol mannosides. We did not find differences in the presence of these glycolipids, but a deeper analysis of the cell wall composition of each mycobacterium in each medium is currently being undertaken. Moreover, alteration in the cell wall composition modifies cell wall permeability, probably also leading to a variation in antigen release. Therefore, not only lipidic antigens but also released proteins are modified in the same species after growth in different culture media [11,15,56]. Curiously, each commercially available *M. bovis* BCG strain is grown in different conditions containing different nitrogen and carbon sources, which ultimately may affect its efficacy [57]. Discerning the specific antigenic profile of each mycobacterium in each culture medium could provide us with information about the unknown antigens needed for antitumoral activity.

Our study highlights the relevance of biotechnological production of mycobacterial biomass. In addition to the slow fastidious growth of some species compromised by the use of enriched media, the intrinsic hydrophobicity and clumping behavior of mycobacteria in liquid cultures limits the use of bioreactors for mycobacterial mass production. This problem can be resolved by adding detergents to the liquid media, such as Tween or Tyxolapol [32], which facilitate the disruption of mycobacterial aggregates, thereby improving their growth. However, these detergents could remove some lipidic and glycolipidic compounds from the surface of the mycobacteria, modifying their antigenic properties and hence altering the interaction with the host [58,59,60,61]. Consequently, static liquid cultures such as those described here are still used. 

Further research is warranted to find the best system either using solid, liquid cultures or bioreactors to improve the reproducibility and quality controls in mycobacteria biomass production. This is especially urged when rough mycobacteria, which grow forming clumps in liquid media like *M. bovis* BCG or *M. brumae,* are used. Our present work highlights the relevance of selecting the most appropriate compounds and/or concentration of culture medium to address this challenge. The composition of the culture medium in which mycobacteria grow could affect the immunogenic profile of the outer cell wall altering the immunogenic profile of the mycobacterial cell surface or modifying the capacity of secretion of non-structural immunogenic antigens. Next experiments in in vivo models should be also carried out to corroborate the impact of culture media composition on the immunomodulatory and antitumoral effect of mycobacteria.

## 5. Conclusions

In the present work, we demonstrated that the culture medium formulation directly influences the antitumoral and immunomodulatory capacity of mycobacteria and is specific for each species. Moreover, these culture conditions are not necessarily those in which high mass production is obtained. Although the results observed in these in vitro experiments seem to be relevant for the immunotherapeutic use of mycobacteria in cancer treatment, in vivo studies are required to confirm these findings. Our results provide insight into the antitumor activity of mycobacteria in cancer research and indirectly into the meticulousness required for mycobacterial production.

## Supplementary Materials

The following are available online at https://www.mdpi.com/2076-2607/8/5/734/s1.

## Appendix A

Mycobacterium genera has recently divided in four different new genera. *Mycobacterium brumae* is officially denominated *Mycolicibacterium brumae*.
