# Supplementary material for: Each Mycobacterium Requires a Specific Culture Medium Composition for Triggering an Optimized Immunomodulatory and Antitumoral Effect
Source: Microorganisms. 2020 May 14;8(5):734. doi: 10.3390/microorganisms8050734 (PMC7284523; doi:10.3390/microorganisms8050734)
Supplement: Supplementary file 1 [file microorganisms-08-00734-s001.pdf]

microorganisms

MDPI

Article

Each Mycobacterium Requires a Specific Culture Medium Composition for Triggering an Optimized Immunomodulatory and Antitumoral Effect

Sandra Guallar-Garrido[1], Víctor Campo-Pérez[1,2], Alejandro Sánchez-Chardi[3,4], Marina Luquin[1] and Esther Julián[1,*]

Table S1. Formulations of Sauton medium present in literature.

	Sauton (1912)	Boyden *et al.* (1954)	Kusunose *et al.* (1976)	Chadwick *et al.* (1982)	Harth *et al.* (1997)	Petricevich *et al.* (2001)	Petricevich *et al.* (2001)	Batista *et al.* (2004)	Larsen et al. (2007)	Mehra et al. (2014)	Li *et al.* (2014)	Teknova
**L-asparagine (g/L)**	4	6	4	4,54		4	-		4	4	1	4
**Sodium glutamate (g/L)**							4					
**L-glutamate (g/L)**					2						2 to 8	
**Soluble starch (g/L)**								1				
**Bacto-peptone (g/L)**	-	-	-	-	-	-	-	16.6				-
**Glycerol (mL/L)**	60	30	53.3	60	60	60	30	60	60	60	2	17.8
**Zinc sulphate (mg/L)**	-	-	-	-	-	-	-		1	1		-
**Sodium-potassium phosphate (g/L)**						0.5		0.5				
**Citric acid (g/L)**	2	2	2	2	2	2.41	2	2.41	2	2	2	2
**Potassium phosphate (g/L)**	0.5	1.5	0.5	0.5	0.5		0.5		0.5	0.5	0.5	0.5
**Ferric ammonium citrate (g/L)**	0.05	0.05	0.05	0.05	0.05	0.495	0.05	0.495	0.05	0.05	0.05	0.05
**Magnesium sulphate (g/L)**	0.5	0.25	0.5	1	0.5	0.5	0.5	0.5	0.5	0.5	0.5	0.5
**Tween 80 (mL/L)**		-	-	-		-	-		0.5	0.5	0.5	0.15
**Glucose (g/L)**	-	10	-	-	-	-	-					2
**pH**	7.4	6.2	7.1	7.2	7.4	7.2-7.25	7	7.2-7.25	7	7		7

Table 2. Colony forming Units (CFU) counts obtained from the innoculum used for infection experiments.

Culture media	**CFU/mL of *M. brumae* innoculum**									
	**A60**		**G15**		**G60**		**7H9**		**7H10**	
Infected cells	Average	Range	Average	Range	Average	Range	Average	Range	Average	Range
**MB49**	7.88E+05	5.50E+05 - 1.40E+06	1.06E+06	6.75E+05 - 1.85E+06	7.38E+05	5.50E+05 - 9.50E+05	8.13E+05	3.75E+05 - 1.25E+06	7.92E+05	5.50E+05 - 1.18E+06
**5637**	5.63E+05	4.50E+05 - 7.25E+05	9.63E+05	4.00E+05 - 1.85E+06	7.00E+05	4.50E+05 - 8.75E+05	7.96E+05	4.25E+05 - 1.25E+06	5.92E+05	4.50E+05 - 8.75E+05
**T24**	1.06E+06	4.50E+05 - 2.05E+06	8.79E+05	4.00E+05 - 1.85E+06	5.83E+05	3.75E+05 - 8.25E+05	7.38E+05	4.25E+05 - 1.25E+06	5.17E+05	3.75E+05 - 6.25E+05
**SW780**	1.21E+06	5.50E+05 - 2.05E+06	1.00E+06	4.00E+05 - 1.85E+06	5.83E+05	3.75E+05 - 7.00E+05	8.25E+05	4.25E+05 - 1.18E+06	9.00E+05	3.75E+05 - 1.70E+06
**J774**	1.00E+06	4.95E+05 - 1.79E+06	4.43E+05	3.25E+05 - 5.75E+05	4.33E+05	3.45E+05 - 5.30E+05	5.26E+05	3.45E+05 - 6.30E+05	1.56E+06	1.28E+06 - 1.79E+06
**THP-1**	6.31E+05	1.80E+05 - 1.45E+06	6.59E+05	2.30E+05 - 1.27E+06	3.62E+05	1.50E+05 - 6.30E+05	4.28E+05	1.90E+05 - 8.25E+05	1.64E+06	3.70E+05 - 3.54E+06

Culture media	**CFU/mL of *M. bovis* BCG innoculum**									
	**A60**		**G15**		**G60**		**7H9**		**7H10**	
Infected cells	Average	Range	Average	Range	Average	Range	Average	Range	Average	Range
**MB49**	5.42E+05	4.00E+05 - 7.00E+05	5.67E+05	3.75E+05 - 7.25E+05	6.21E+05	4.50E+05 - 7.00E+05	5.75E+05	4.50E+05 - 7.50E+05	5.29E+05	4.00E+05 - 7.25E+05
**5637**	8.67E+05	4.00E+05 - 1.50E+06	7.19E+05	6.25E+05 - 9.50E+05	9.79E+05	4.75E+05 - 1.48E+06	5.50E+05	4.50E+05 - 7.25E+05	6.21E+05	4.75E+05 - 8.50E+05
**T24**	6.25E+05	3.75E+05 - 1.33E+06	8.56E+05	6.25E+05 - 9.50E+05	7.55E+05	3.75E+05 - 1.10E+06	4.96E+05	4.25E+05 - 5.50E+05	5.88E+05	3.75E+05 - 8.50E+05
**SW780**	8.00E+05	3.75E+05 - 1.50E+06	1.14E+06	4.25E+05 - 2.23E+06	9.35E+05	3.75E+05 - 1.48E+06	9.54E+05	4.25E+05 - 2.00E+06	4.96E+05	3.75E+05 - 7.25E+05
**J774**	5.98E+05	3.00E+04 - 1.86E+06	6.50E+05	4.00E+04 - 1.86E+06	6.20E+05	8.50E+04 - 1.86E+06	1.03E+06	1.20E+05 - 1.65E+06	9.96E+05	1.70E+05 - 1.95E+06
**THP-1**	2.69E+05	9.50E+04 - 4.15E+05	3.10E+05	8.00E+04 - 6.25E+05	1.18E+05	8.00E+04 - 1.65E+05	1.54E+06	1.00E+05 - 3.85E+06	1.38E+06	1.80E+05 - 3.30E+06

Data is the average of CFU/mL from a duplicate culture of the inoculum of three independent experiments of infection

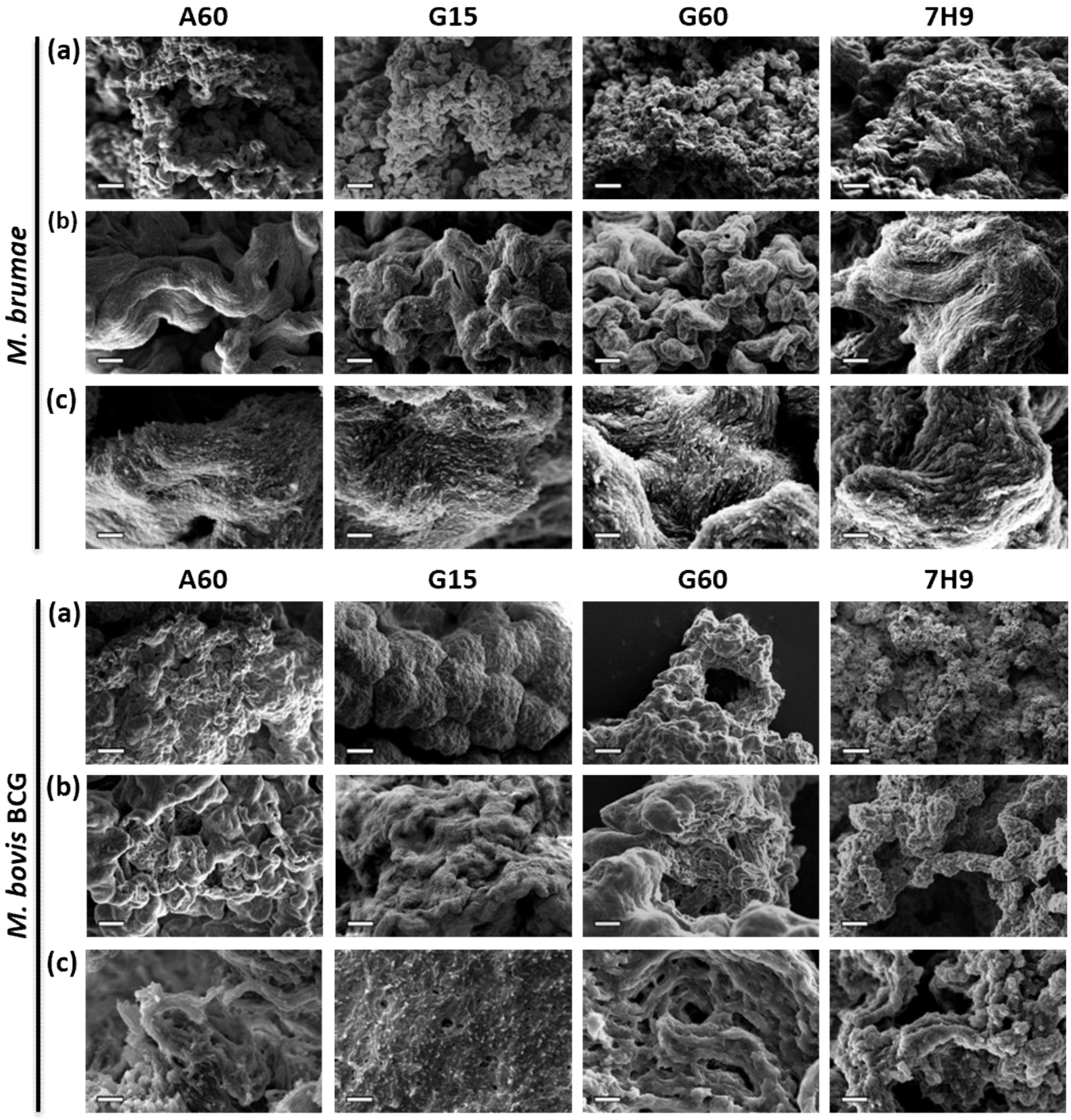

Supplementary Figure S1. Representative SEM micrographs of *M. brumae* and *M. bovis* BCG pellicles grown on optimized Sauton media. (a) Low-magnification images showing the general surface appearance of the pellicles, scale bars correspond to 130 μm. (b) Medium-magnification images showing structures formed by the ordered distribution of mycobacteria into the pellicle, scale bars correspond to 30 μm. (c) High-magnification images showing the detail of the bacilli that form the pellicle, scale bars correspond to 6 μm.

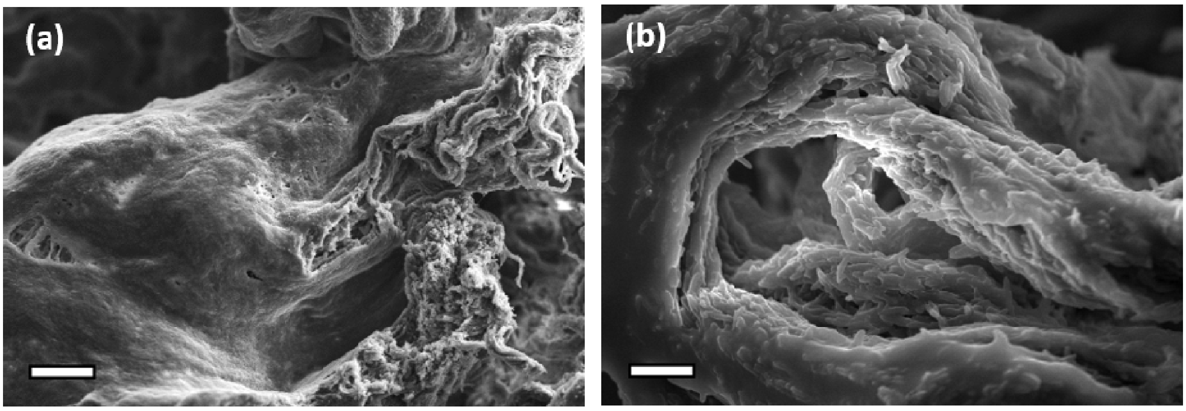

Supplementary Figure S2. Detail of *M. bovis* BCG grown in A60 Sauton media. Scale bars correspond to 30 μm (a) and 6 μm (b).
